# Prevalence and determinant factors of premenstrual syndrome among secondary and preparatory school students in Dessie city administration, Northeast Ethiopia

**DOI:** 10.1186/s12905-024-03219-4

**Published:** 2024-06-24

**Authors:** Demissie Teshome Wochekaw, Dagnachew Melak, Yonas Fissha Adem, Tesfalem Tilahun Yemane, Shambel Ayichew Tsegaw, Mengistu Mera Mihiretu

**Affiliations:** 1 Department of Public Health, Dessie Health Science College, Dessie, Ethiopia; 2 Department of Epidemiology and Biostatistics, School of Public Health, Colleges of Medicine and Health Science, Wollo University, Dessie, Ethiopia; 3 Department of Health System Management, School of Public Health, College of Medicine and Health Sciences, Wollo University, Dessie City, Ethiopia

**Keywords:** Premanstrual syndrom, Determinants, Adolescent school girls, Dessie, Northeast Ethiopia

## Abstract

**Background:**

In Ethiopia, premenstrual syndrome (PMS) was predominantly studied among university students who were in their early 20s; as a result, little is known about the prevalence of premenstrual syndrome among adolescent girls. Therefore, this study aimed to determine the prevalence of premenstrual syndrome and identify factors associated with premenstrual syndrome among secondary school female students in the Dessie city administration, 2023.

**Methods:**

An institutional-based cross-sectional study was conducted involving a sample of 630 participants. A structured self-administered data collection tool was used to gather the necessary information. To ensure data quality, the pretesting and training of the data collectors and supervisors were conducted. The collected data were entered into Epi-data software and analyzed using SPSS version 25. Frequency tables, graphs, means, and medians were used to describe the characteristics of the study participants. Binary logistic regression was employed to identify significant factors. Variables with a *p*-value ≤ 0.05 with 95% confidence interval (CI) of adjusted odds ratio (AOR) in the final multivariable logistic regression were reported as statistically significant factors associated with PMS. Model fitness was evaluated using the Hosmer and Lemeshow goodness-of-fit test.

**Results:**

In the present study, the prevalence of PMS was 22%, 95% CI = 19-26%. Factors: Age ≥ 18 years (AOR = 0.54; 95% CI: 0.34, 0.86), duration of menstruation ≥ 7 days (AOR = 3.61; 95% CI: 1.25, 10.37), presence of chronic illness (AOR = 2.08; 95% CI:1.04, 4.16), coffee intake (AOR = 6.05; 95% CI: 2.05, 17.87), alcohol intake (AOR = 0.49; 95% CI: 0.28, 0.86), use of pain medication (AOR = 2.06; 95% CI:1.10, 3.86), use of hormonal contraceptives (AOR = 3.9; 95% CI:1.58, 9.62), sleep disturbance (AOR = 3.82; 95% CI: 2.29, 6.42) and physical exercise (AOR = 0.50; 95% CI: 0.28, 0.87) were significantly associated with PMS.

**Conclusion:**

A significant number of students in this study were affected by premenstrual syndrome. Age, duration of menstruation, presence of chronic illness, coffee intake, use of pain medication, use of hormonal contraceptives, and sleep disturbance were significantly associated with PMS. Students should avoid excessive use of alcohol, coffee intake and use of pain medication without prescription.

## Introduction

Numerous women experience physical or mood changes during menstruation [1]. Premenstrual syndrome (PMS) is characterized by a recurring, cyclical, and predictable set of cognitive, psychological, and somatic symptoms occurring during the luteal phase [2, 3]. These symptoms manifest consistently each month throughout the menstrual cycle, impacting a woman’s regular daily activities [1]. The underlying pathophysiology of PMS, which is multifactorial and complex, is unclear, and factors such as hormonal changes, diet, and lifestyle may play roles [4].

The American College of Obstetricians and Gynecologists (ACOG) criteria are commonly utilized for the diagnosis of premenstrual syndrome (PMS) [1]. These criteria delineate cyclical psychological and physical symptoms, with a severity threshold requiring identifiable social or economic dysfunction [1]. In each of the three preceding menstrual cycles, at least one affective symptom (such as depression, angry outbursts, irritability, anxiety, confusion, or social withdrawal) and one somatic symptom (such as headache, swelling of extremities, breast tenderness, or abdominal bloating) must be reported within five days before menstruation [1, 2].

Premenstrual syndrome affects women of childbearing age, particularly those in their late 20s to mid-40s, and is a significant public health concern for young girls [4, 5]. Depending on the stringency of diagnostic criteria, PMS affects approximately 25% of women with regular menstrual cycles [2]. The prevalence of PMS, as studied by the Royal College of Obstetricians and Gynecologists (RCOG), reveals that 4 out of 10 women experience premenstrual symptoms, with 5–8% severely affected by PMS [6]. A systematic review and meta-analysis (SRMA) conducted in France indicated the lowest rate at 12%, while studies in Iran reported the highest rate at 98% [7]. PMS is also prevalent in Turkey, particularly among women in the younger age group, with reported prevalence rates ranging from 66–91.8% [8–10]. In Ethiopia, studies among Jimma University students reported the lowest prevalence of PMS (27%) [11], whereas research among Addis Ababa University students indicated the highest prevalence (84%) [12].

Although the exact cause of PMS is unclear, elevated estrogen levels, decreased progesterone levels, and heightened aldosterone hormone activity may contribute to PMS [13]. Previous studies have indicated various factors linked to PMS, including nutritional status, contraceptive pill usage, social and cultural influences, lack of exercise, psychological stress, family history, and lifestyle [14]. Additionally, factors such as body mass index (BMI), pain medication use, caffeine consumption [15], family history of PMS, hormonal contraceptive use [16], and early menarche [17] are identified as potential contributors to PMS.

Many women and clinicians view PMS as a normal and non-distressing syndrome [18, 19], with only 28.8% seeking medical help due to attitudinal barriers, despite its serious impact on physical and mental health [20]. PMSs are strongly linked to poor physical health, increased healthcare use, reduced quality of life, and psychological suffering, which affect work productivity, daily activities, and social interactions, including sexual relationships [21]. It is recognized as a public health problem affecting women, families, societies, and nations [21].

Despite global studies highlighting the prevalence and impact of PMS, limited attention has been given to this issue in developing countries such as Ethiopia [21]. Existing studies in Ethiopia primarily focus on university students in their early 20s, mostly assessing severe forms of PMS, leaving a gap in understanding the prevalence of PMS among adolescent girls in secondary and preparatory schools, especially in the study area [12, 22–25] As a result, little is known about the prevalence of PMS among adolescent girls attending in secondary and preparatory schools, particularly in this study area.

This study provides context-based evidence for determining the prevalence of PMS and identifying associated factors in secondary and preparatory schoolgirls. Therefore, this study aimed to assess the prevalence of PMS and identify factors associated with PMS among secondary and preparatory schoolchildren in the Dessie city administration, 2023.By addressing the lack of information on the prevalence of premenstrual syndrome (PMS) and associated factors among high school students in the study area, this study aimed to serve as a valuable reference for healthcare providers.

## Methods and materials

### Study setting, period, and study design

The research was conducted in secondary and preparatory schools in Dessie city administration, located approximately 400 km from Addis Ababa, Ethiopia. It took place from April 20 to May 15, 2023, using an institution-based cross-sectional study design.

### Study participants

The study focused on the entire population of female students enrolled in grades 9 to 12 within secondary and preparatory schools in the Dessie city administration. This included all regular female students attending both public and private schools. All female students in grades 9 to 12, whether in governmental or nongovernmental schools, were considered in the study. The exclusion criteria for the study were female students who faced difficulties speaking, hearing, or seeing; who were unwell during the data collection period; and who were attending night programs.

### Sample size determination and sampling technique

The sample size for the study was determined using both the single population proportion formula and the double population formula with statistical calculation software. The calculation was based on a pooled incidence of 53.8% for PMS in Ethiopia [17] with the assumptions of a 95% confidence interval (CI), a 5% margin of error, and a design effect of 1.5. The single population proportion formula resulted in a larger sample size, specifically 382. Accounting for a potential nonresponse rate of 10%, a total of 630 students were selected to participate in the study.

n=$$\frac{{Za/2}^{2}\text{*} \text{P} (1-\text{P})}{{W}^{2} }$$

*P* = 53.8%, W = 0.05, Z = 1.96 (for 95% CI).

n= $\frac{{1.96}^{2}\text{*}0.538\left(0.462\right)}{{0.05}^{2} }$=382; for a nonresponse rate of 10%, the final sample size was 382*10%=37.6 ~ 38, 382+38) *1.5=630 participants

To calculate the sample size by the second objective, Epi info version 7 was used to analyze factors associated with PMS in previous similar studies. The 95% confidence level (CL), 80% power, and 1:1 ratio of exposed to unexposed individuals were assumed, and factors such as family history of premenstrual syndrome, amount of menstrual bleeding, BMI, age at menarche, duration of menses and history of sexual intercourse were considered from a previous similar study (Table 1). The maximum sample size was 214 according to the duration of menses [26].

**Table 1:** Sample size calculation for the second objective of studying the prevalence and determining factors of PMS among secondary and preparatory school students in the Dessie city administration, 2023

Factors	References	% PMS in exposed	% PMS in unexposed	AOR	95%CI for AOR	Total Sample size
**Family history of PMS** Exposed (Yes) Unexposed (No)	[16]	75	32.3	6.34	(3.47,11.56)	50
**amount of menstrual bleeding** Exposed (Moderate) Unexposed (Mild)	[16]	66	19	8.55	(2.78,26.33)	40
**BMI (kg/m2)** < 18.5 (Exposed) 18.5–24.9 (unexposed)	[12]	81	59	3.01	(1.71–6.3)	148
**Age at menarche (years)** 13–15 16–18	[12]	20	4	6.12	(1.99–19.8)	148
**Duration of menses** 1–3 days (Unexposed) > = 7 days (Exposed)	[26]	21	7	3.58	(1.53, 8.37)	214
**History of intercourse** Yes (unexposed) No (exposed)	[27]	21	5	5.03	(2.20,11.51)	162

Dessie city administration has 10 secondary schools with a current enrollment of 8014 female students. Using a lottery method, four schools (30% of the total) were selected. A sampling frame was created by compiling a list of all female students, along with their grades and sections, from each selected school. The sample size was proportionally allocated, and individual participants from each grade level and section in the chosen schools were randomly selected using a computer-generated simple random sampling method.

### Study variables

The dependent variable was the presence of premenstrual syndrome (yes/no). The independent variables included socio-demographic factors such as age in years, religion, parents’ employment status, marital status, parents’ occupation, and academic grade level.

Lifestyle and behavioral factors included smoking history, daily coffee consumption in the Arabian cup, alcohol consumption, use of pain relievers, use of sleeping pills, use of hormonal contraceptives, history of sexual intercourse, and physical activity level.

The menstruation-related variables included age at menarche, pattern of menses, duration of bleeding in days, amount of menstrual flow, length of the menstrual cycle, history of depression, and presence of chronic illness. The anthropometric and food intake variables included height in meters, weight in kilograms, use of fried foods, and use of sweet drinks and food.

### Operational definition

Premenstrual syndrome (PMS) - Participants who scored twenty or more points, equivalent to 50% of the total 40 questions on the PMS scale, experienced symptoms occurring approximately four days before menstrual flow and lasting until the onset of bleeding. The severity of PMS increases with increasing score [28].

The scoring system used “never” as “1,” “rarely as “2,” “sometimes as “3,” “very often as “4,” and usually as “5”. The percentage scores were used to categorize symptoms as “no symptoms” (1–40), “mild” (41–80), “moderate” (81–120), “severe” (121–160), or “very severe” (161–200) [28]. Premenstrual dysphoric disorder (PMDD) is a severe form of PMS characterized by prominent psychological and behavioral symptoms such as labile and depressed mood, anger, irritability, and internal tension [25]. Mild PMS involves symptoms not interfering with routine daily activities (41–80 PMS symptoms), moderate PMS includes symptoms impacting routine daily activities (81–120 PMS symptoms), and severe PMS entails symptoms hindering participation in any activity (≥ 121 PMS symptoms) [26, 28]. Coping mechanism: Participants reported taking any action to alleviate or reduce premenstrual symptoms, regardless of their severity. Participants scoring above 50% on the 15 questions assessing coping mechanisms were considered to have a history of coping [26, 28].

### Data collection tool and data quality control

A self-administered structured questionnaire was employed for data collection and was developed through a comprehensive review of various studies, with modifications based on insights gathered during a rigorous pretest, and a reliability test was conducted using Cronbach’s alpha test (scale reliability coefficient of 0.8275).

The questionnaire covered socio-demographic variables, lifestyle and behavioral factors, menstrual-related aspects, and anthropometric and dietary conditions [14–17]. The ACOG premenstrual syndrome screening tool, which consists of 40 symptoms, was used to measure PMS, which was categorized as either “yes” or “no” [28].

The data collection was carried out by eight trained female teachers who are Bachelor of Science (BSc) and Bachelor of Art (BA) degree holders, and the tool was carefully designed and translated into the local language (Amharic). A pretest involving 5% of the total sample (21 students) at Kombolcha Millennium School led to important modifications. Two degree female teachers from each school served as data collectors, with one supervisor assigned to each school to oversee and manage the overall data collection process. Both the data collectors and supervisors underwent a three-day training session covering the steps, procedures, and proper handling techniques for data collection.

### Data processing and analysis

The data were examined for completeness and coded before entry. A template was created in Epi-data version 4.6 software for data entry, and SPSS version 25 was utilized for data analysis. Following data cleaning, descriptive measures such as frequencies and percentages for categorical variables and means and medians for continuous variables were calculated for all variables related to the study objectives. The results are presented in tables, text, and graphs to depict the characteristics of the study participants.

Simple binary logistic regression analysis (bi-variable binary logistic regression) was applied for each independent variable with the dependent variable. All independent variables with a *P* value < 0.2 in the bi-variable analysis were included in the final multivariable analysis. Determinant factors with an adjusted odds ratio (AOR) and a *P* value < 0.05 at the 95% confidence interval in the multivariable analysis were considered statistically significant determinants of PMS. The variance inflation factor (VIF) and tolerance were used to assess multi-collinearity, and to check for model fitness, the goodness-of-fit test of Hosmer and Lemeshow was employed.

## Results

### Socio-demographic characteristics of participants

A study was conducted with the participation of 617 students, for a response rate of 98%. Nearly all of the participants (615 individuals, 99.7%) had never been married, and the majority of the participants (253 individuals, 41%) were in Grade 12. The average age of the participants was 17 (± 1.37) years. In terms of the employment status of the student’s parents, 366 individuals (59.3%) had employed parents, with 257 individuals (41.7%) working in the government sector (Table 2).

**Table 2:** Socio-demographic characteristics of students in Dessie Preparatory and High School, Dessie, Northeast Ethiopia, 2023

Variables	Category	Number (*n*)	Percentage (%)
Marital status	Single	615	99.7
	Married	2	0.3
Age in year	≤ 17 years	330	53.5
	≥ 18 years	287	46.5
Religion	Orthodox	309	50.1
	Muslim	303	49.1
	Protestant	2	0.3
	Others	3	0.5
Educational status	Grade 9	87	14.1
	Grade 10	48	7.8
	Grade 11	229	37.1
	Grade 12	253	41
Employment Status of parent	Employment	366	59.3
	unemployment	251	40.7
Parent occupation	Government worker	257	41.7
	Private worker	112	18.2
	Merchant	144	23.3
	Farmers	71	11.5
	Others	33	5.3

### Menstrual-related characteristics of participants

The average age at which menstruation began was 13 ± 1.29 years, and the majority of participants (503 individuals, 81.5%) experienced their first period after the age of 13. Four hundred ninety-two participants (79.7%) reported having a regular menstrual cycle. In terms of the length of the menstrual cycle, 202 participants (32.7%) reported a cycle length of fewer than 21 days, while 31 participants (5%) reported a cycle length of more than 35 days. Out of all the respondents, 59 individuals (9.6%) reported a menstrual flow duration of 1–2 days, while 92 individuals (15%) reported more than 7 days. Additionally, 38 participants (6%) reported a history of sexual intercourse (Table 3).

**Table 3:** Menstrual-related characteristics of Dessie preparatory and high school student participants, Dessie, Northeast Ethiopia, 2023

Variables	Category	Number (*n*)	Percentage (%)
Age at menarche	≤ 12 years	114	18.5
	≥ 13 years	503	81.5
Menstrual Cycle Pattern	Regular	492	79.7
	Irregular	125	20.3
Menstrual cycle length in days	< 21	202	32.7
	21–35	384	62.2
	> 35	31	5
Duration of menstrual flow in days	1–2	59	9.6
	3–6	466	75.5
	>=7	92	14.9
Amount of menstrual flow	Heavy	97	15.7
	Normal	421	68.2
	Light	99	16
History of sexual Intercourse	Yes	38	6.2
	No	579	93.8
History of depression	Yes	131	21.2
	No	486	78.8
History of chronic illness	Yes	53	9.2
	No	560	90.8

### Dietary condition and anthropometric measurements of the respondents

Among the respondents, 290 individuals (47%) reported frequent consumption of fried foods, while 281 individuals (45.5%) reported consuming sweet foods and drinks. The average weight of the respondents was 46 ± 6.15 years, and the average height was 1.52 ± 0.19 m. In terms of body mass index (BMI), 202 respondents (32.7%) had a BMI ≤ 18.4 kg/m2, 367 respondents (59.5%) had a BMI of 18.5–24.9 kg/m2, and 46 respondents (7.5%) had a BMI ≥ 25 kg/m2.

### Lifestyle- and behavioral-related characteristics of the respondents

Of the total participants, 14 students (2.3%) reported a history of cigarette smoking, while 499 students (80.9%) had a regular or occasional history of alcohol consumption. Additionally, 85 students (13.8%) reported using pain relievers, and 33 students (5.3%) reported using hormonal contraceptives. Sleep disturbance was reported by 210 students (34%), and 20 students (3.2%) reported using sleeping pills. In terms of physical activity, 78 students (12.6%) engaged in regular physical activity, while 418 students (67.7%) engaged in occasional physical activity. Concerning nonacademic activities within their households, 59 students (9.6%) were involved in heavy activities, while 462 students (74.9%) were engaged in simple nonacademic activities with their families (Table 4).

**Table 4:** Lifestyle- and behavioral-related characteristics of the participants among students in Dessie Preparatory and High School, Dessie, Northeast Ethiopia, 2023

Variable	Category	Number (*n*)	Percentage (%)
Smoking history	Yes	14	2.3
	No	603	97.7
Coffee intake in cups per day	0	477	77.3
	1–4	117	19
	> 5	23	3.7
Tea intakes per day	Yes	400	64.8
	No	217	35.2
Drinking alcohol	Yes	499	80.9
	No	118	19.1
Use of pain relief	Yes	85	13.8
	No	512	86.2
Use of sleeping pill	Yes	20	3.2
	No	597	96.8
Use of hormonal contraceptives	Yes	33	5.3
	No	584	94.7
Sleep disturbance	Yes	210	34
	No	407	66
Physical activity	Regularly	78	12.6
	Occasionally	418	67.7
	Not at all	121	19.6
Involvement in non academic duty in the family	Not at all	96	15.6
	Simple	462	74.9
	Heavy	59	9.6

### Prevalence of premenstrual syndrome in Dessie preparatory and high school students

This study revealed that the prevalence of premenstrual syndrome (PMS) among preparatory and high school students in Dessie city administration was 22.4%, with a 95% confidence interval of 19-26%. To assess PMS, three sets of questions were used, which included questions concerning physiological symptoms (16 symptoms), psychological symptoms (12 symptoms), and behavioral symptoms (12 symptoms), adopted from the American Cooperative Oncology Group (ACOG). Physiological symptoms were reported by 38 participants (6.2%), while psychological and behavioral symptoms were experienced by 66 participants (11%) and 31 participants (5.2%), respectively. In terms of coping mechanisms for PMS, an average of 108 respondents (18%) reported using various strategies, such as resting, consuming hot drinks, engaging in physical exercise, and seeking healthcare.

### Factors associated with premenstrual syndrome

According to the bi-variable binary logistic regression analysis, several factors were found to be associated with PMS. These factors included the age of the participants, the pattern of their menstrual cycle, the length and duration of their menstrual flow, the amount of menstrual flow, the presence of chronic illness, a history of smoking, coffee, and alcohol intake, the use of sleeping pills and pain relievers, the use of hormonal contraceptives, sleep disturbance, and physical exercise. These associations were significant at the 0.2 level.

According to the multivariate binary logistic regression analysis, several factors were significantly associated with PMS at the 0.05 level of significance. These factors included the age of the participant, the duration of menstruation in days, the presence of chronic illness, coffee intake, the use of pain relievers, the use of hormonal contraceptives, and sleep disturbance.

Participants aged 18 years and older had 46% lower odds of experiencing PMS than those aged 17 years and younger (AOR = 0.54; 95% CI = 0.34, 0.86). A duration of menstruation of more than 7 days increased the odds of having PMS by 3.61 times compared to a flow duration of 1–2 days (AOR = 3.61; 95% CI = 1.25, 10.37). Students with chronic illness were 2.08 times more likely to experience PMS than students without chronic illness (AOR = 2.08; 95% CI = 1.04, 4.16). Daily coffee intake of more than 5 cups was positively associated with PMS (AOR = 6.05; 95% CI = 2.05, 17.87). Alcohol intake reduced the odds of PMS by 51% (AOR = 0.49; 95% CI = 0.28, 0.86). The use of pain relievers increased the odds of PMS by 2.06 times (AOR = 2.06; 95% CI = 1.10, 3.86), while the use of hormonal contraceptives (AOR = 3.9; 95% CI = 1.58, 9.62) and experiencing sleep disturbance (AOR = 3.82; 95% CI = 2.29, 6.42) were also positively associated with PMS (Table 5).

**Table 5:** Bivariate and multivariate binary logistic regression analyses of factors associated with PMS among high school and preparatory students in Dessie City, Northeast Ethiopia, 2023

		Premenstrual syndrome (PMS)				
Factors	Category	Yes	No	COR (95%CI)	AOR (95%CI)	*P* value
Age in year	≤ 17 years	83 (13%)	247 (40%)	1	1	
	≥ 18 years	55 (9%)	232 (38%)	0.71(0.48,1.04)	0.54(0.34,0.86)	**0.010**
Menstrual cycle	Regular	90 (15%)	402 (65%)	1	1	
	Irregular	48 (8%)	77 (12%)	2.78(1.82,4.27)	1.37(0.78, 2.41)	0.273
Menstrual cycle length in days	≤ 21 days	47 (7%)	155 (25%)	1	1	
	21–35 days	80 (13%)	304 (49%)	0.87(0.58,1.31)	0.81(0.49,1.34)	0.408
	> 35 days	11 (2%)	20 (3%)	1.81(0.81,4.06)	2.26(0.79,6.42)	0.126
Duration of menstrual flow in days	1–2 days	9 (1%)	50 (8%)	1	1	
	3–6 days	94 (15%)	372 (60%)	1.40(0.67,2.96)	1.33(0.53,3.36)	0.547
	≥ 7 days	35 (6%)	57 (9%)	3.41(1.50,7.79)	3.61(1.25,10.37)	**0.017**
Amount of menstrual flow	Heavy	36 (6%)	61 (10%)	2.66(1.38,5.12)	2.14(0.95,4.84)	0.066
	Normal	84 (14%)	337 (55%)	1.12(0.64,1.97)	1.40(0.71,2.75)	0.327
	Light	18 (3%)	81 (13%)	1	1	
Presence of chronic illness	Yes	31 (5%)	26 (4%)	5.05(2.88,8.86)	2.08(1.04,4.16)	**0.038**
	No	107 (17%)	453 (73%)	1	1	
History of smoking	Yes	6 (0.9%)	8 (1%)	2.68(0.91,7.85)	1.3(0.35.65)	0.727
	No	132 (21%)	471 (76%)	1	1	
Coffee intake	No intake	85 (14%)	392 (64%)	1	1	
	1–4 cup/day	44 (7%)	73 (12%)	2.78(1.79,4.32)	3.67(2.12,6.35)	**< 0.001**
	≥ 5 cup/day	9 (1%)	14 (2%)	2.97(1.24,7.07)	6.05(2.05,17.87)	**0.001**
Alcohol intake	Yes	92 (15%)	407 (66%)	0.35(0.23,0.55)	0.49(0.28,0.86)	**0.012**
	No	46 (7%)	72 (12%)	1	1	
Use of sleeping pills	Yes	10 (2%)	10 (2%)	3.66(1.49,9.01)	2.23(0.65,7.66)	0.203
	No	128 (21%)	469 (76%)	1	1	
Use of pain medication	Yes	33 (5%)	52 (8%)	2.58(1.50,4.19)	2.06(1.10,3.86)	**0.023**
	No	105 (17%)	427 (69%)	1	1	
Use of hormonal contraceptive	Yes	18 (3%)	15 (2%)	4.64(2.27,9.48)	3.9(1.58,9.62)	**0.003**
	No	120 (19%)	464 (75%)	1	1	
Sleep disturbance	Yes	76 (12%)	134 (22%)	3.16(2.14,4.66)	3.82(2.29,6.42)	**< 0.001**
	No	62 (10%)	345 (56%)	1	1	
Physical exercise	Regularly	26 (4%)	52 (8%)	1.01(0.55,1.85)	0.60(0.28,1.26)	0.177
	Occasionally	72 (12%)	346 (56%)	0.42(0.27,0.67)	0.50(0.28,0.87)	**0.014**
	Not at all	40 (6%)	81 (13%)	1	1	

## Discussion

This study revealed that the prevalence of PMS was 22.4%, 95% CI: 19-26%. These findings were in line with those of studies of Jimma University students [11] and those of Zagazig University, Egypt [29].

However, these findings were lower than those of studies conducted across the world, such as a study in Saudi Arabia [30], a study performed in Lebanon [15], and a study in Brazil [31]. These findings are also lower than those of similar studies conducted in specific areas of Ethiopia; for example, a study in Mekelle showed that 30.9% of respondents had PMS [25]. A study of Addis Ketema high school students showed that 86.1% of the patients reported having PMS during their lifetime [21], and a systematic review and meta-analysis (SRMA) was conducted in Ethiopia [17].

The prevalence of PMS may appear to be relatively low. There are several explanations for the low prevalence of PMS in certain contexts; for instance, the discrepancy may be attributed to differences in population characteristics, as Premenstrual syndrome is more common among individuals in their late 20s to mid-40s [4, 5], and limited awareness and knowledge about PMS among healthcare providers and the general population can also contribute to under-diagnosis and underreporting. Women may not recognize their symptoms as related to PMS or may attribute them to other causes, leading to a lower prevalence rate in this study.

These findings are somewhat greater than those of systematic reviews and meta-analyses in France [7]. This difference could be due to the inconsistency of the diagnostic criteria for PMS. These findings were also greater than those of a study conducted on preparatory school students in Hawassa [24]. PMS symptoms can vary widely in terms of severity and manifestation. Some women may experience mild symptoms that do not significantly impact their daily lives, leading to a lower likelihood of seeking medical attention or reporting their symptoms.

In this study, the age of the participants was significantly associated with PMS; age > 18 years was negatively associated with PMS. These findings were consistent with those of previous studies in Ambo University, Debre Markos town, and Aligarh. Uttar Pradesh [4, 22, 27] showed that young age was associated with PMS. PMS is believed to be influenced by hormonal fluctuations, particularly changes in estrogen and progesterone levels during the menstrual cycle [4]. As women age, their hormone levels naturally fluctuate and decline. These hormonal changes can impact the occurrence and severity of PMS symptoms. Therefore, age-related hormonal shifts may contribute to the association between age and PMS symptoms. It is worth noting that while age has been associated with PMS, individual experiences can vary significantly. Not all women experience PMS symptoms, and the severity and duration of symptoms can differ among those who experience PMS. However, further research is needed to better understand the complex relationship between age and PMS symptoms and to explore the potential underlying mechanisms involved.

A duration of menstruation for more than seven days was positively associated with PMS symptoms; these findings were consistent with those of studies in Egypt, Aligarh, Uttar Pradesh, Wolkite, Mekelle, and Debre Markos, which revealed that a longer duration of menstruation was positively associated with PMS [4, 25–27, 29]. Different explanations can be attributed to this association; longer menstrual bleeding can result in increased inflammation in the reproductive system. The prolonged presence of menstrual blood and tissue can lead to an inflammatory response, which may contribute to the severity of PMS symptoms such as bloating, breast tenderness, and mood swings [32].

The presence of chronic illness was positively associated with PMS in this study, which was consistent with the findings of a study in Turkey [33]. Individuals with chronic illnesses may have heightened sensitivity to physical and psychological changes in their bodies, and other medications and treatments used to manage chronic illnesses can have an impact on hormonal balance and overall well-being, which collectively increase the likelihood of experiencing PMS symptoms.

Another contributing factor to PMS in this study was coffee intake, which was positively associated with PMS. This finding was supported by the findings of a study at Ambo University, Ethiopia, Lebanon, and Ankara, Turkey [15, 22, 33]. Coffee intake, especially in high amounts, may interfere with serotonin levels, potentially exacerbating PMS symptoms. Several studies suggest that caffeine may also interfere with fluid balance in the body, leading to increased fluid retention. This fluid retention can worsen bloating and breast tenderness, which are common PMS symptoms [34]. The ACOG recommended that women who experience PMS should avoid caffeine consumption [1]. However, numerous studies have not found substantial evidence linking caffeine consumption to premenstrual syndrome (PMS). Even when comparing women with the highest and lowest caffeine intakes in these studies, there was no significant positive association between consuming high amounts of caffeinated coffee and experiencing PMS or specific symptoms such as breast tenderness [35].

The use of pain medication, hormonal contraceptives, and sleep disturbance were other significant factors associated with PMS. Frequent use of pain medication increases the likelihood of PMS; this finding was congruent with the findings of a study performed in Lebanon [15]. Pain medication is usually taken to reduce menstrual-related discomfort; frequent use of this medication may lead to exacerbation of symptoms of PMS by altering hormone levels.

The use of hormonal contraceptives also increases the likelihood of PMS; this finding is supported by a similar study in Bahirdar town [16]. Hormonal contraceptives, such as combined oral contraceptives (COCs) or hormonal patches, contain synthetic hormones that regulate the menstrual cycle. This hormonal regulation can lead to a reduction in the severity of PMS symptoms or even prevent them altogether.

However, the effects of hormonal contraceptives on PMS can vary among individuals. While some individuals may experience a reduction in PMS symptoms with the use of hormonal contraceptives, others may not notice a significant change or may even experience an exacerbation of symptoms. This implies that it is crucial to consult with a healthcare professional to discuss the potential benefits and risks of using hormonal contraceptives for managing PMS symptoms. They can provide personalized guidance based on individual health conditions and preferences.

### Strengths and limitations of the study

This study uses relatively a large sample size by considering a sufficient number of schools in the study area; thus it has better generalizability of the findings. The limitation of this study was the use Cross-sectional study design makes it difficult to ascertain the cause-effect relationship of independent and dependent variables. Self-report information from the study participants and absence of a confirmatory mechanism for PMS symptoms may under or overestimate the prevalence of PMS.

## Conclusion

A significant number of students in this study were affected by PMSs. However, the prevalence of PMS in this particular study was relatively lower than that in other similar studies conducted in specific areas of Ethiopia. Age, duration of menstruation in days, presence of chronic illness, coffee intake, use of pain relievers, use of hormonal contraceptives and sleep disturbance were significantly associated with PMS. Students should avoid frequent use of pain medications and hormonal contraceptives. Students should reduce excessive coffee consumption by more than 5 cups/day, as excessive coffee consumption disturbs the balance between estrogen and progesterone, leading to the appearance of PMS symptoms.

## Data Availability

Raw data for dataset D1 are not publicly available to preserve individuals’ privacy under the European General Data Protection Regulation.
